# Somatovisceral Convergence in Sleep-Wake Cycle: Transmitting Different Types of Information *via* the Same Pathway

**DOI:** 10.3389/fnetp.2022.840565

**Published:** 2022-03-02

**Authors:** Ekaterina Levichkina, Marina L. Pigareva, Alexandra Limanskaya, Ivan N. Pigarev

**Affiliations:** ^1^ Department of Optometry and Vision Sciences, The University of Melbourne, Parkville, VIC, Australia; ^2^ Institute for Information Transmission Problems (Kharkevich Institute), Russian Academy of Sciences, Moscow, Russia; ^3^ Institute of Higher Nervous Activity and Neurophysiology, Russian Academy of Sciences, Moscow, Russia

**Keywords:** somatovisceral convergence, interoceptive network, spinal cord, sleep-wake cycle, lemniscus pathway, referred pain, neural networks in sleep

## Abstract

Convergence of somatic and visceral inputs occurs at the levels of nervous system ranging from spinal cord to cerebral cortex. This anatomical organization gave explanation to a referred pain phenomenon. However, it also presents a problem: How does the brain know what information is coming for processing—somatic or visceral - if both are transferred by the same spinal cord fibers by means of the standard neuronal spikes? Recent studies provided evidence for cortical processing of interoceptive information largely occurring in sleep, when somatosensation is suppressed, and for the corresponding functional brain networks rearrangement. We suggest that convergent units of the spinal cord would be able to collectively provide mainly somatosensory information in wakefulness and mainly visceral in sleep, solving the puzzle of somatovisceral convergence. We recorded spiking activity from the spinal cord lemniscus pathway during multiple sleep-wake cycles in freely behaving rabbits. In wakefulness high increased spiking corresponded to movements. When animals stopped moving this activity ceased, the fibers remained silent during passive wakefulness. However, upon transition to sleep fibers began firing again. Analysis of spiking patterns of individual fibers revealed that in the majority of them spiking rates recovered in slow wave sleep. Thus, despite cessation of motion and a corresponding decrease of somatic component of the convergent signal, considerable ascending signaling occurs during sleep, that is likely to be visceral. We also recorded evoked responses of the lemniscus pathway to innocuous electrostimulation of the abdominal viscera, and uncovered the existence of two groups of responses depending upon the state of vigilance. Response from an individual fiber could be detected either during wakefulness or in sleep, but not in both states. Wakefulness-responsive group had lower spiking rates in wakefulness and almost stopped spiking in sleep. Sleep-responsive retained substantial spiking during sleep. These groups also differed in spike amplitudes, indicative of fiber diameter differences; however, both had somatic responses during wakefulness. We suggest a mechanism that utilizes differences in somatic and visceral activities to extract both types of information by varying transmission thresholds, and discuss the implications of this mechanism on functional networks under normal and pathological conditions.

## Introduction

Convergence of somatic and visceral afferent inputs to the same neurons at the level of spinal cord was discovered in 19th century, yet its functional meaning remains a mystery. This bizarre arrangement of signal transmission, where the same neuronal fiber sends to the brain signals coming from functionally different systems, is implicated to be responsible for so called referred or reflected pain, when visceral disturbances are felt as pain in somatic areas not necessarily adjacent to its source, as e.g., a heart attack perceived as neck, shoulder or back pain (for the review see Giamberardino et al., 2006). However, somatovisceral convergence is incredibly rarely discussed beyond the scope of the subject of referred pain, and the role of this system in normal functions is unknown.

For obvious reasons, no study of somatovisceral convergence can apply all potentially possible types of visceral stimulation in a single experiment, and usually studies are restricted to one or two stimulation types that are reasonably easy to apply in a controlled manner. The types of visceral stimulation most often used are either mechanical distension of the distensible organs or electrical stimulation of the nerves expected to carry a signal of interest to the spinal cord. For example, Honda (1985) studied single unit responses to the bladder or colon distension and to somatic stimulation in cat sacral spinal cord in the area surrounding central canal, and described the following proportion of the response types: 48% selectively somatic, 6% selectively visceral and 46% converging somatovisceral. Al-Chaer et al. (1999) reported similar proportion of dorsal column and spinothalamic fibers (43 and 41% correspondingly) responsive to colon distension in macaques. Cervero and Tattersall (1986) studied neuronal responses to the splanchnic nerve stimulation in the thoracic spinal cord, and found 56–75% of them to be affected by that stimulation in addition to their somatic inputs, with some neurons being selectively somatic, while no purely visceral responses were observed.

Although the role of somatovisceral convergence in referred pain has been extensively discussed (Cervero and Tattersall, 1986; Giamberardino, et al., 2006), we know from experience that signals from distensible organs such as urinary bladder or colon usually induce behavior leading to their voiding well before the sensations become noxious. These convergent signals do not only participate in transmitting noxious sensation, but are also likely to reflect normal activity of visceral systems. For example, the role of the dorsal column in transmission of the innocuous visceral signals has been pointed out by Al-Chaer et al. (1999).

Considering high proportion of the “converged” units, responsive to just one type of visceral stimulation, it seems safe to assume that the actual degree of convergence, if all visceral signals could be taken into account, might be even higher than the individually reported 40–75% rates. Convergent signaling can be traced from the pre-spinal level up to the thalamus and finally brain cortex (Langford and Coggeshall, 1981; Pierau et al., 1984; Cervero and Tattersall, 1986; Brüggemann et al., 1994; Brüggemann et al., 1997; Al-Chaer et al., 1999). Thus, ascending signals would quite normally represent a mixture of two seemingly unrelated physiological responses. That raises the question: How does the brain make sense of this information?

It is important to note that exploration of somatovisceral convergence has been often conducted in anaesthetized animals, which results in an estimation of responses that cannot be reliably broken down by state of vigilance, and the degree of similarity between natural activity of the spinal cord and the one produced in these experiments is also largely unknown. State of vigilance strongly affects movements—muscle tone is diminished in slow wave sleep (SWS) and progresses to muscle atonia in REM (Chase, 2013), which in turn is likely to change the somatic component of spinal cord spiking activity. What happens to the visceral part of the converged somatovisceral signal during transition from wakefulness to sleep remains an open question. At the same time, parasympathetic system becomes more active in sleep (Trinder et al., 2001; Cabiddu et al., 2012), visceral organs remain active as well, and a range of bodily regulatory events occur selectively in sleep (Moore, 1991; Besedovsky et al., 2012; Ali et al., 2013) which leads us to an assumption that visceral component of the converging signal may be preserved or even enhanced in SWS. In this case sleep-related cessation of the somatic signal would clarify the visceral part of the convergent input, possibly making it more accessible for transmission and analysis in the brain. If this signal clarification indeed takes place, then the brain receives “pure” visceral signals via spinal cord in a regular fashion, every sleep-wake cycle in sleep, that can promote development of sleep-specific networks involved in visceral regulation in the brain, as suggested by the *visceral theory of sleep* (Pigarev, 2014; Pigarev and Pigareva, 2015). Brain network re-formation upon transition from wakefulness to sleep has been indeed reported in humans (Larson-Prior et al., 2011; Tarun et al., 2020). Once the network is expanded to include muscle activity and some visceral parameters apart from just the brain, it becomes clear that network alterations occur between sleep stages as well, as demonstrated using recently developed time delay stability (TDS) method (Bashan et al., 2012; Ivanov, 2021).

It is also known that ascending signals from the spinal cord produce synchronizing effect upon the brain, while lesions or blockage of spinal cord decrease EEG slow-wave activity (Hodes, 1964; de Andrés et al., 1976; de Andrés et al., 2011). Similar effects can be induced by afferent vagus nerve input (Puizillout et al., 1973; Valdés-Cruz et al., 2008). Thus, some component of spinal activity seems likely to be associated with sleep and to promote sleep in a similar way to the largely visceral vagal input. We have recently reported that neurons of cat insular cortex, the area involved in viscerosensory and visceromotor processing, are three times more likely to respond to innocuous visceral stimulation in SWS in comparison to wakefulness (Levichkina et al., 2021), indicating that processing of visceral signals is enhanced in sleep. Moreover, the majority of insular cells responding in sleep do not respond during wakefulness and vice versa, demonstrating a possibility of segregation between networks in different states of vigilance. Neurons of the monkey primary somatosensory cortex which exhibit somatovisceral convergence also demonstrate dependency of responses from the level of anesthesia (Brüggemann et al., 1997). Recent study has demonstrated enhanced cortical responses to vagal stimulation across multiple cortical areas including somatosensory and motor cortices during SWS in comparison to wakefulness or REM sleep (Rembado et al., 2021), as earlier predicted (Pigarev et al., 2020). At the same time somatic component of the response—cortical somatosensory evoked potential to innocuous stimuli—is reduced in SWS (Shaw et al., 2006). Thus, there is a clear dynamics of enhancement of the visceral signaling accompanied by a diminishing of the somatic one upon transition from wakefulness to sleep. These changes are consisted with the observations of studies conducted within the Network Physiology framework, which reported decoupling of the brain-muscle network nodes during deep sleep and REM (Bashan et al., 2012; Balagué et al., 2020; Rizzo et al., 2020). There is a growing body of evidence for a widespread involvement of brain cortex in viscerosensation and visceromotor regulation, especially for the somatosensory/motor areas (for the review see Azzalini et al., 2019), which makes this dynamics an important issue for understanding the brain component of visceral dysregulations, and especially its relations to sleep and sleep disturbances. These data highlight the importance of studying interactions between brain and various visceral systems in different physiological states, as this approach can be instrumental in revealing deviations from optimal parameters of visceral regulation leading to a disease (Bashan et al., 2012; Kenett et al., 2014; Ivanov, 2021).

In this article we explored general pattern of activity in the lemniscus pathway of dorsal spinal cord across sleep-wake cycle as, to the best of our knowledge, there is no description of that transition in the available literature. If both converging signals were only relevant to wakefulness-related signal processing, one would expect the spike rates in the pathway to drop progressively from active wakefulness to quiet wakefulness and then decrease further in slow wave sleep. However, if only the somatic component is depressed in SWS, a substantial proportion of cells receiving converging input would remain active, as visceral signals would still be transmitted.

Considering that for some of the visceral signals it is necessary to be perceived consciously to guide behavior, while the others are necessary for visceral regulation but do not usually invade conscious perception, we hypothesized that convergent somatovisceral signals in the spinal cord can also occur in two groups of cells, one responding to visceral stimuli predominantly during wakefulness and the other during sleep (SWS).

To test the above mentioned hypothesis of the functional separation of signals between wakefulness and sleep, we also studied neuronal responses to innocuous electrical stimulation of the abdominal viscera in wakefulness and sleep within that pathway. The experiments were conducted using freely behaving rabbits. High degree of similarity in the organization of the ascending spinal pathways and their targets exists in mammals, and in fact it appears on the evolutionary tree even earlier as such similarity exists between reptiles and mammals as well (Ebbesson and Goodman, 1981). It is therefore likely for the mechanisms of somatovisceral convergence to be conservative between species as well, with the obvious inclusion of humans.

On the basis of our findings we suggest a simple model of processing that can make use of the revealed types of convergent signals and is capable of deciphering both somatic and visceral types of information from it.

## Materials and Methods

The experiments were conducted on five freely behaving grey rabbits (Chinchilla strain, 3–3.5 kg body weight). First experiment was focused on detailed description of neuronal activity within lemniscus pathway during sleep-wake cycle. To achieve that, electrophysiological recordings were made across multiple cycles with recording electrodes remaining at the same position for the whole duration of the experiment. In this experiment minimal number of transitions between wakefulness and SWS was 9, and the maximal number was 39, with at least 50 s of active wakefulness when the animal was moving, followed by at least 200 s of motionless with a transition to SWS (judged by cortical activity and somnographic parameters). This experiment involved four out of five rabbits, total number of recorded units was 143, numbers per rabbit are provided in Table 1.

**TABLE 1:** Distribution of cell activity types during the transition from active wakefulness to SWS.

Rabbit	N cells recorded	N no return	N half return	N complete return
1	37	5	14	18
2	40	14	12	14
3	35	3	15	17
4	31	0	14	17
Total, 4 animals	143	22 (15.4%)	55 (38.5%)	66 (46.1%)

The second experiment aimed to explore evoked responses to innocuous electrical stimulation of abdominal viscera across sleep-wake cycle and involved two rabbits. Electrical stimulations were balanced between SWS and wakefulness, and after disregarding the intervals containing artifacts and the ones when stimulations occurred during transition between sleep and wakefulness, the mean numbers of stimulation in slow wave sleep and wakefulness were equal (mean N = 51, SD in sleep = 20, SD in wakefulness = 23). Positions of the recording electrodes were changed between stimulation sessions in order to accumulate data with the amounts of individual units suitable for further analysis of evoked responses (total N = 135, of them 109 recorded from rabbit 2 and 26 from rabbit 5 correspondingly).

Rabbits are crepuscular animals with a predisposition to have more SWS and REM sleep during the light part of the cycle; however they have many short sleep-wake cycles during both light and dark phases (Tobler et al., 1990). To obtain data from both wakefulness and sleep recordings were made during daytime in Experiment 2 and both daytime and nighttime in Experiment 1.

Surgery and treatment of the animals were carried out in accordance with the Ethical Principles for the maintenance and use of animals in neuroscience research (Zimmermann, 1987) and NIH guidelines for the care and use of animals. In accordance to Russian regulations, assessment of the ethical components of a research proposal was conducted first by the Council of Reviewers prior to making a decision regarding financial support of a study, and then by the Institutional Scientific Council. Both Councils are guided by the recommendations of the above-mentioned documents.

The animals’ movements were unrestricted, and a rabbit’s hopping can be quite vigorous, which is why a major issue limiting experiment duration was a decrease of recording quality due to a recording electrode damage/connection loss. Duration of recording period therefore ranged from 1 week to 3 months.

Recordings of dorsal spinal cord activity were made at the thoracolumbar level of the spinal cord (T12–L4 segments), from 1 to 4 recording electrodes per animal. To assess state of vigilance the following somnographic parameters were co-recorded as well: electroencephalogram (EEG), electromyogram of the neck muscles (EMG), electrocardiogram (ECG), and breathing rate (BR), accompanied by video recording.

### Surgery

Rabbits are not required to fast before surgery; on the contrary, it is important to ensure uninterrupted gastrointestinal (GI) activity in these animals, otherwise recovery after surgery can be impeded. This requirement limits a safe duration of surgery as GI activity can be impaired by anesthesia. Therefore implantation of recording electrodes was split into two procedures: the first included implantation of electrodes required for polysomnography (EEG and EMG), and during the second one spinal cord recording platform was implanted and recording electrodes placed (see details below). The surgeries were separated by at least 1 week recovery period, and the same duration recovery period was implemented between the surgery and the onset of recordings. To ensure an appropriate GI system recovery the animals were fed before each surgery and monitored afterwards; the rabbits were expected to start eating within 2 h after recovery from anesthesia (motions onset). If eating onset was delayed, GI-stimulating i.m. injection of metoclopramide was made (Cerucal, 0.5 mg/kg). Rabbits were pre-operatively sedated with xylazine (Xyla, 23.3 mg/ml, 0.15 ml/kg); meloxicam was used for analgesia (0.3 mg/kg). Surgeries were conducted under Zoletil anesthesia (equal parts of tiletamine hydrochloride and zolazepam hydrochloride) that was initially administered in a relatively small dose to avoid renal complications (7.5 mg/kg), and was topped up during surgery when required, supplemented by local infiltration with Lidocaine (2–4 mg/kg at surgical site). Enrofloxacin antibiotic was injected during the procedures (10 mg/kg).

### Implantation of EEG and EMG Electrodes

Soft tissues were removed from the dorsal surface of the skull and five blunt screws made of an orthopedic grade stainless steel inserted epidurally. Two of them were placed over left and right parietal areas for EEG recordings, one used as a ground for EMG recording and the rest served as anchoring screws to improve implant stability. Two hook electrodes for bipolar EMG recordings were introduced into the neck muscle using a hypodermic needle. The EMG electrodes were made of 0.5 mm lacquered constantan wires with insulation removed from the tips, exposing 2 mm of metal. A recording socket was formed and the skull surface covered with Vertex acrylic dental cement.

### Implantation of the Spinal Recording Platform and Electrodes Placement

Skin above two adjacent vertebrae was cut medially, above the spinous processes, and muscles over the right side of the vertebrae retracted to expose dorsal surface next to the spinous processes. 2 mm wide bone openings for recording were drilled at the base of the processes obliquely towards the midline of the spinal cord, and the additional openings for ground wires made through the spinous processes horizontally. We aimed to record from several electrodes simultaneously (up to 4), placed at the level of one or of two adjacent vertebrae, to increase chances of getting a signal from at least one electrode after animal’s recovery, since connection integrity was a common issue and not all the electrodes remained intact across the whole duration of the experiment. The intended positioning of the electrodes tips were the dorsal column-medial lemniscus pathway (DCML), however due to small size openings we were not able to further guide visually the electrode placement and therefore refrain from specifying the position in relation to the midline. For the first experiment requiring prolonged recordings we used stainless steel Teflon insulated wires (Cooner wire) inserted subdurally, but without deep penetration of the neural tissue. The electrode tip was cleared of insulation and sharpened using a micro-grinder, and internal 1 mm part of the electrode was pre-bent to be introduced in an angle to the surface of the spinal cord, and intended to penetrate only the dura, the electrodes were not repositioned in this experiment. The placement was guided by the first appearance of neuronal spikes in the recorded signal. If inserted further, the electrodes might damage spinal cord tissue over the time required for data collection in multiple sleep-wake cycles.

For Experiment 2 electrodes needed to be repositioned to increase the number of recorded units, thus we inserted robust tungsten varnish-coated microelectrodes via plastic conical guiding tubes and moved them using miniaturized custom-made micromanipulators (electrode manufacturing and many of the related implantation techniques were described in detail in Pigarev et al., 2009; Levichkina et al., 2021). However, protrusion of the electrodes was restricted to 1 mm superficial part of the pathway to avoid tissue damage related to animal’s movements. In both experiments bone surfaces surrounding the drilled openings were covered with acrylic dental cement, a small protecting platform was made over the exposed vertebrae using steel wires covered by dental cement, and the soft tissues sutured around the dental cement platform and guiding tubes. In both experiments electrodes had 0.5–1 mΩ impedance.

### Electrical Stimulation of the Abdominal Viscera

Stimulation was applied via two constantan Teflon-coated wires injected bilaterally into the ventral half of the abdominal cavity using hypodermic needles (the procedure is similar to a regular intraperitoneal injection), one at the level of 1st and the other at the level of 6th lumbar vertebrae, using the following stimulation parameters: 1–2 mA current, 0.6–1 m s duration; it was applied every 40 s in rabbit 2 and every 60 s in rabbit 5. Before commencing a stimulation session the parameters above were adjusted in a way that the animal did not show any behavioral response to stimulation (no postural change/individual muscles visibly twitching, and no signs of increased alertness/discomfort). As the current was passed between the two wires, it was expected to travel diagonally through the abdominal viscera, activating various nerves, and local neuronal networks. Thus, in comparison to stimulation of a single nerve, the response to such stimulation might be delayed, complex and prolonged, since different visceral processes with their own response times can be evoked. Although that approach seems less controllable in comparison to nerve stimulation, it is also less traumatic to an animal as no additional surgery would have been required to implant cuff electrode for nerve stimulation.

### Data Acquisition

All recording cables were connected to a slip ring connector placed above the animal’s enclosure to allow it to move freely. Ad lib food and water access was provided during and between recording sessions.

Signals were amplified (Neurobiolab) and recorded to a hard drive for off-line analysis using either PowerLab or Spike 2 acquisition systems. The analogue filter for EEG and BR recording was set for 1–50 Hz, for EMG and ECG for 1–1,000 Hz, and for spinal cord either 0.3–3,000 or 10–3,000 Hz. Sampling rates for EEG, ECG, and BR recordings was 200 Hz, for EMG 1,000 Hz, and for the spinal cord 10,000 Hz. Spinal cord activity was high-pass filtered at 250 Hz, and neuronal spikes produced by different units were sorted by the Spike 2 inbuilt algorithm using both amplitude triggering and waveform matching, then spikes from different neurons were stored and analyzed individually. Spiking events were smoothed with Gaussian kernel of µ = 20 ms (Wallisch et al., 2010), and transformed into the spike frequency rate curves. We further refer to the recorded units as “cells” for simplicity since the spiking is actually produced by the cells, however considering the anatomy it is more likely that spiking activity was actually recorded from the fibers (axons) rather than the cell bodies.

Recordings were visually inspected to identify periods of wakefulness, SWS, and REM based on polysomnographic data and behavioral observation (presence or absence of motion, position of the ears, nose twitching etc.), periods containing artifacts resulted from too vigorous movements of an animal that led e.g., to a saturation of any of the recorded signals were excluded from further analysis.

### Data Analysis

#### Experiment 1: Activity of the Lemniscus Pathway During Sleep-Wake Cycle

We marked time points when active wakefulness that lasted at least 50 s stopped for at least 200 s, these time points are further called “transition points”. All further analysis was conducted using custom-made Matlab scripts.

**FIGURE 1:**
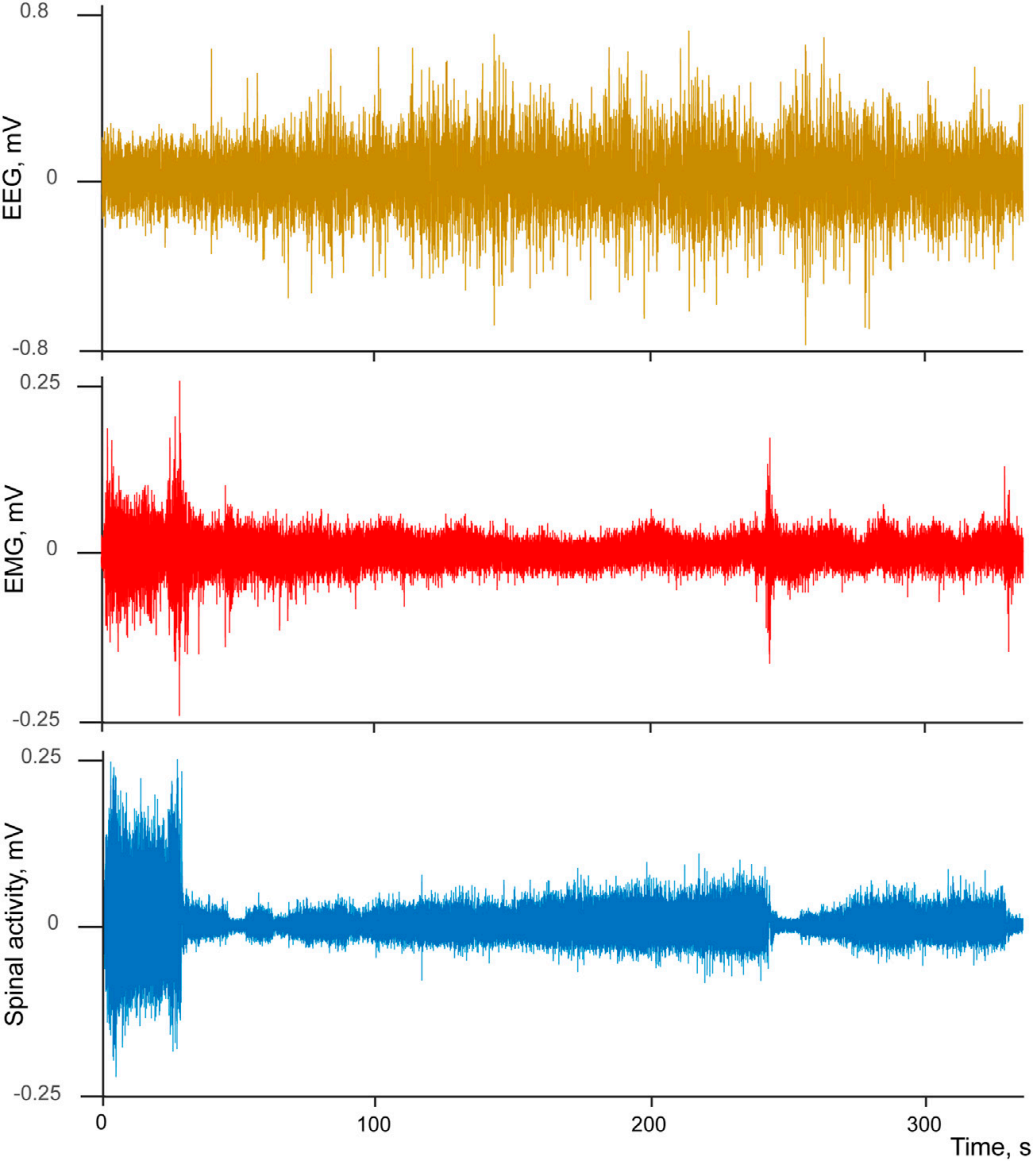
Example of simultaneously recorded EEG (brown), EMG (red), and spinal cord (blue) activities during transition from wakefulness to sleep.

To visualize the state of vigilance we used EEG signal bandpass-filtered in delta range (1–4 Hz, Butterworth two order bidirectional filter) and then obtained its envelope (absolute value of the Hilbert transform). Neuronal activity and the above-described envelope of EEG delta component were aligned using the transition points as triggers, and later averaged. We noticed that multiunit neuronal activity often demonstrated a characteristic pattern across sleep-wake cycle. Intense spiking corresponding to the animal’s movement was drastically reduced at the transition from active to passive wakefulness, but increased again during wakefulness-to-sleep transition. This pattern is exemplified by Figure 1 which demonstrates raw EEG, EMG and spinal cord signals for a single interval containing active movements at the beginning, then passive wakefulness transitioning to sleep, and then again a short interval of movements following by falling asleep. One can see a characteristic “lying Christmas tree” pattern of the spinal cord activity emerging during the transition between states of vigilance.

We studied activities of the individual cells across this cycle to identify whether all cells had similar patterns or only a fraction of them was responsible for the observed effect of “spiking return” during sleep. In order to do that we calculated averaged activity surrounding maximal response during active period of wakefulness (mean spike rate at ± 5 ms interval) and used it as a measure of activity restoration during sleep. Within 200 s after cessation of movements for every cell we calculated: latency of spike rate minimum (Lmin), latency of the spike rate returning to half of it maximal value (Lhalf), and latency of the spike rate becoming equal to its maximal value (Lmax). We assessed stability of the effects by statistically comparing spike rates around the activity maximum in wakefulness to the activity minimum upon transition to quiet wakefulness and to the activity maximum when activity in sleep reached the same level as in active wakefulness for all cells where this effect was found (Wilcoxon signed-rank test, *p* < 0.05).

All neurons sending their axons *via* the lemniscus pathway are expected to reflect somatic responses. To test that, we calculated Pearson correlation between EMG signal and spike rates of the individual cells across the entire period of analysis (covering all sleep-wake cycles). Both signals were averaged in a sliding window of 250 ms, with 5 ms step.

#### Experiment 2: Lemniscus Pathway Evoked Responses to Visceral Stimulation in Sleep-Wake Cycle

10 ms long intervals immediately following every stimulation were excluded from the analysis as they contained stimulation artifacts.

Responses to stimulation were averaged separately for the stimulation applied during wakefulness and slow wave sleep. Response significance was established using Reed et al. criteria (2011), where to be considered significant the response had to deflect from pre-stimulus background (500 ms before the stimulation onset) by at least three standard deviations (SD), and remain consistently deviated from that baseline by at least two SD for at least 40 ms within 10–510 ms interval after the onset of stimulation. Our choice of long post-stimulus interval for analysis was in part informed by the overall durations of spinal cord evoked potentials to stimulation of peripheral visceral nerves (Selzer and Spencer, 1969), as even direct nerve stimulation produced complex evoked potentials lasting for at least 150 ms, and our type of stimulation can potentially cause slow or long-lasting visceral effects, which can in turn be reflected in delayed spinal activity. For six cells responses were observed beyond 510 ms, within 1 s after stimulus onset. As the potential duration of the visceral changes is unknown, for these cells we extended both background and response intervals and set them up at 1 s, applying the same response criteria.

It has been reported earlier that fibers within the pathway might have different diameters and myelination (Thomas et al., 1984), which can be reflected in their spike amplitudes. Thus we decided to measure amplitudes of the averaged spikes for every cell that passes the above mentioned response criteria, as a magnitude of the extremum of the extracellular potential deflection (AMP, described in detail in Mosher et al., 2020). That was done to estimate whether those cells, which are sensitive to visceral stimulation in wakefulness could be distinguished from the ones responding in sleep.

We also tested whether responding cells indeed always had strong somatic component by calculating Pearson correlation between EMG and spike rates as described for Experiment 1, and estimated mean background activity for each cell in wakefulness and sleep to assess the changes of spike rate between states.

In both Experiments all statistical comparisons of the reported parameters between groups of cells were done using Wilcoxon signed rank test in the case of paired comparisons, and Wilcoxon rank sum test for the unpaired ones, and *p* < 0.05 used as the criteria for the significance of a difference between groups.

## Results

### Activity of the Lemniscus Pathway During Sleep-Wake Cycle

**FIGURE 2:**
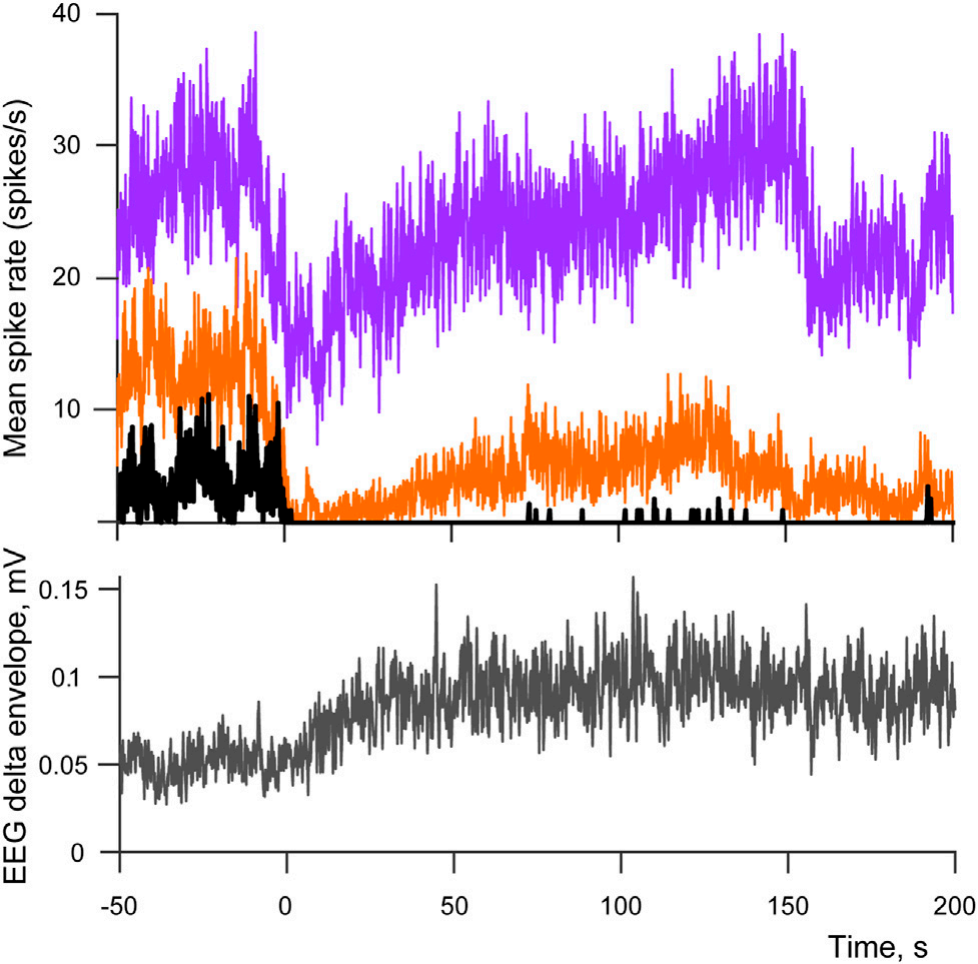
Spiking patterns across sleep-wake cycle. Top panel demonstrates averaged spike rates of three cells belonging to “no return” (black) “half return” (orange) and “complete return” (purple) types of spiking activity. Bottom panel shows the corresponding averaged envelope of EEG delta activity. 0 indicates the moment when movements stopped after a period of active wakefulness (transition points used as triggers for averaging). The number of averaged sleep-wake cycles is equal to 17.

We studied spiking patterns of 143 individual cells recorded from four rabbits across multiple sleep-wake cycles (ranging from 9 to 39 transition points per recording session). The majority of cells (87 out of 143, *p* < 0.05) had their response minima within the interval immediately following a cessation of movements. Although in only 87 cells this spiking rate drop reached significance, the effect is likely to be more widespread as in many cases the absence of a significant difference was caused by high variance of the cell’s spiking during movements rather that the absence of a drop afterwards. Mean latency of the drop (Lmin) was equal to only 12.1 s (SD = 11.7 s) from the end of the last EMG deviation from its mean, thus the spiking minimum likely occurred well before sleep had a chance to develop, and for the majority of cells it happened during passive wakefulness, and for the rest of the cells during early sleep. A consequent recovery of spiking was observed during SWS, with three types of neuronal activity present: 1) selectively wake-active cells that during sleep never return to the level of spiking rate observed in active wakefulness—“no return” type, 15.4% of all recorder cells, 2) cells that return to or exceed that level—“complete return” type, 46.1%, and 3) cells that reached spiking rates above half of the active wakefulness level but stayed below the level of active wakefulness—“half return”, 38.5%. Thus most of the cells had a complete or partial recovery of spiking activity during SWS. Mean latency of half-return—Lhalf was equal to 68.5 s (SD = 58.3 s), and the complete return of spiking to its highest level Lmax occurred at 61.8 s (SD = 57.7 s). The timelines of spiking recovery were therefore similar for “half return” and “complete return” types. Examples of neuronal activity for these types of cells from one recording session are demonstrated on Figure 2 together with the averaged envelope of EEG delta component for the same recording, one can see the enhancement of delta-activity, which accompanies development of sleep. The numbers of cell responses per type and rabbit are provided at Table 1.

135 cells out of 143 demonstrated significant positive correlations between EMG and spiking rate, thus the majority of cells had some degree of somatic responsiveness, ranging from mild to high (*p* < 0.005, mean Rho for all cells with significant positive correlation = 0.26, Rho range from 0.01 to 0.69). Relatively moderate levels of correlation are not surprising as EMG signal was taken from the neck muscles, thus the ideal correspondence between spiking at the thoracolumbar level and cervical EMG cannot be expected. However, the cells that returned to the maximal spiking mode during sleep demonstrated lower correlations with EMG, and cells returning to the half-level had intermediate correlation coefficients correspondingly (mean Rho of “no return” = 0.33, SD = 0.14; mean Rho “half return” = 0.28, SD = 0.12; mean Rho “complete return” = 0.13, SD = 0.19). The “complete return” group also includes the remaining eight cells out of 143, which had low to moderate negative correlation with EMG (Rhos ranging from *−*0.01 to −0.43). Thus, cells resuming activity during sleep might have a considerable degree of activity devoted to a different rather than somatosensory type of signaling, that can potentially be visceral.

### Lemniscus Pathway Evoked Responses to Visceral Stimulation During Sleep-Wake Cycle

**FIGURE 3:**
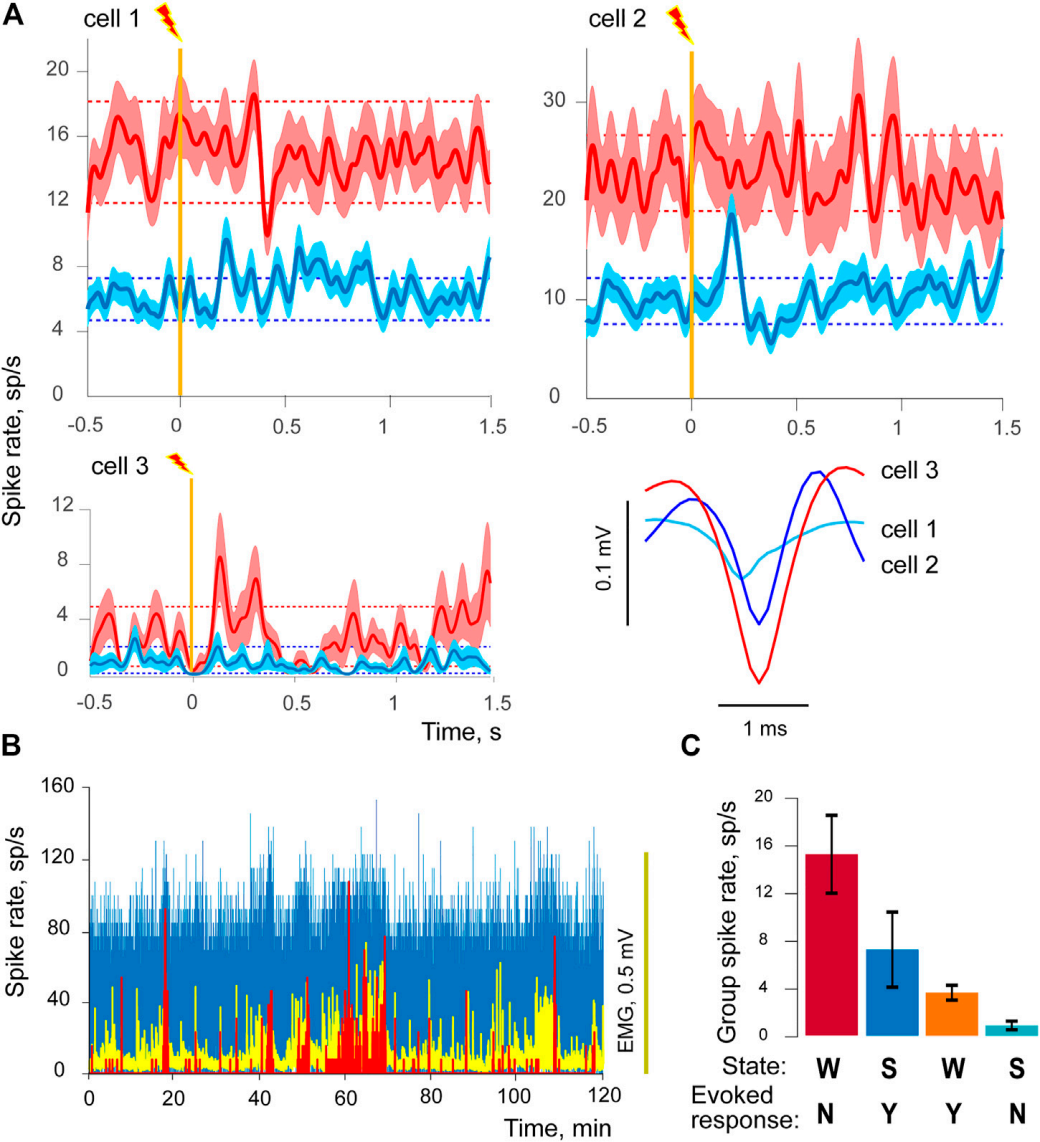
Sleep - responsive and wakefulness - responsive cells. **(A)** Three examples of evoked responses to visceral electrostimulation are given for three cells in wakefulness (red) and sleep (blue), one cell per each of three panels. Solid lines of darker shades demonstrate averaged responses, triggered by stimulation (vertical orange line), light-shaded areas represent standard errors of mean (±SEM). Dashed lines show background-based ±2SD intervals for spike rate. The bottom-right panel of (A) contains the corresponding averaged spike shapes of each of three cells. **(B)** Spike rates of a sleep-responsive cell (in blue), wakefulness-responsive cell (in red), and EMG activity (in yellow) during 2-h long recording session. **(C)** Mean spiking rates (±SEM) of two cell groups in wakefulness and sleep. State of vigilance is indicated by W for wakefulness and S for SWS, presence/absence of the evoked responses to stimulation by Y or N sign. The first two columns from the left side represent the sleep-responsive group, two columns on the right the wakefulness-responsive group of cells.

We analyzed activities of 135 individual units within lemniscus pathway (rabbit 2 N cells = 109, rabbit 5 N = 26). Significant responses to stimulation were detected in 22 cells during wakefulness and in 23 cells during slow wave sleep. Only two cells demonstrated activity in both states, however it is important to note that in both cases responses occurred at different latencies and had opposite polarities in different states (Figure 3A). There was also a significant difference in response type proportions between groups, with sleep-responsive group having only 1 cell with inhibitory response, while 9 out of 22 wakefulness-responsive cells were inhibitory (*p* < 0.05, Fisher exact test).

Another striking effect observed was a pronounced inhibition of baseline activity between wakefulness and sleep in all but four studied cells when background period spike rate was compared between states for each cell individually (*p* < 0.05, Wilcoxon rank sum test). Baseline comparison within each group of cells—wakefulness-responsive or sleep-responsive also resulted in significant differences for both groups (both groups had *p* < 0.001, Wilcoxon rank sum test). However, the effect sizes were different for wakefulness-responsive and sleep-responsive cell types. SWS-related inhibition was considerably less in the group of sleep-responsive cells in comparison to the wakefulness-responsive ones. In order to compare these effects statistically we first calculated an index of background activity (B) change between wakefulness and slow wave sleep (WSI) in the following way:

WSI=(Wakefulness B−SWS B)/(Wakefulness B+SWS B)

WSI is equal to 1 when spiking in sleep ceases completely and is equal to 0 if no change occurs between conditions. We compared WSIs between the groups of sleep-responsive and wakefulness-responsive cells, and the difference was highly significant (*p* = 0.0014). In fact, 10 out of 22 cells from the wakefulness-responsive group demonstrated a complete absence of spiking activity during SWS. In addition to that, background spiking levels in these two groups were also different in wakefulness, namely wakefulness-responsive cells had less background activity. The effect extended to episodes of active movements. We compared mean spike rates obtained from the intervals corresponding to EMG amplitude above its mean, and found that wakefulness-responsive cells demonstrated significantly lower spike rates during active wakefulness as well (mean group spike rate 5.2 for wakefulness-responsive and 13.0 for sleep-responsive cells, *p* = 0.0016). The effect is illustrated for two cells at Figure 3B, where spiking rates of a sleep-responsive cell (in blue) and wakefulness-responsive cell (in red) are shown together with EMG activity (in yellow), the figure demonstrates activities for 2-h long recording session. For illustrative purposes this picture shows cells recorded during the same session, which had extreme spiking differences. One can see that the sleep-responsive cell was active during both high and low EMG activity periods, but the wakefulness-responsive one emitted spikes only when the animal was active and EMG had higher amplitudes. Cells of both groups had significant positive correlations between spiking rates and EMGs, Rhos ranging from 0.1 to 0.57.

Thus, we uncovered that cells belonging to sleep-responsive and wakefulness-responsive groups also demonstrated differences in their general spiking activity (Figure 3, panels (B) and (C)). Sleep-responsive cells had higher frequencies of spiking in both wakefulness and sleep; however both groups had higher spiking rates in wakefulness in comparison to sleep.

Figure 3A provides examples of evoked responses in three different cells along with their averaged spike shapes. Note that all of them demonstrated higher background activity in wakefulness, and the sleep-responsive cells (top row) had higher spike rates in general. Background-based ±2SD intervals are shown as horizontal dashed lines. Cell 1 had prolonged excitatory response in SWS, while its response in wakefulness had much longer latency; it was rather brief and inhibitory. Cell 2 was selectively sleep-responsive, and had two-components, excitatory and inhibitory response in SWS, while no significant response could be detected in wakefulness. Cell 3 was wakefulness-responsive. Wakefulness-responsive cells usually had higher spike amplitudes in comparison to the sleep-responsive ones (*p* = 0.005), which indicates that these groups might also differ in axon diameter, with larger axons in the wakefulness-responsive group Figure 3A (bottom-right panel of Figure 3A).

## Discussion

Our data revealed a pattern of characteristic changes of spiking activity in the lemniscus pathway occurring upon transition from wakefulness to sleep. Spiking drops to its minimal level after cessation of motion, but significantly recovers during transition to SWS. This pattern suggests that some type of activity rather than the attenuated somatosensory one is still present within the pathway during SWS. The degree of correlation between spiking and EMG for the sleep-active cells is also lower than for the cells least spiking during sleep, also indicating that sleep-active cells are less “somatic.” We suggest that recovered activity in sleep represents the other branch of the converged response—the visceral one. Increased responsiveness to visceral events during SWS, that would correspond to increased visceral input in sleep, has been previously reported (Pigarev, 1994; Pigarev et al., 2006; Pigarev et al., 2013; Pigarev et al., 2020; Rembado et al., 2021), and theoretical implications of such switching of inputs was discussed by Pigarev and Pigareva (2014). Previous studies of activity recorded from spinoreticular ascending pathway of the spinal cord also demonstrated preserved spiking during SWS while spiking decreased during REM (Soja et al., 2001), thus relatively high spiking activity in SWS can be common for spinal pathways participating in transmission of both types of information.

Stimulation of the abdominal viscera allowed us to observe two different groups of cells that responded to stimulation selectively in either wakefulness or sleep. These groups also had dramatically different levels of spiking across sleep-wake cycle and differed in the amplitudes of their spikes in a way that suggests that sleep-responsive cells have smaller diameter of axons in comparison to the wakefulness-responsive. A degree of difference in axon diameter within the pathway has been earlier reported for the lemniscus pathway of a cat, with 97% of fibers having axon diameter <8 μm, but 50% being within 2–5 μm range (Thomas et al., 1984; Saliani et al., 2017). Reduced spiking in SWS in both groups of cells indicates partial inhibition of signaling, that we attribute to the decrease of a muscle tone happening during transition from wakefulness to sleep—attenuation of the somatic component of the converging signal (Chase, 2013), since responses of the convergent units are known to have an additive nature, and the largest responses can be elicited by both somatic and visceral types of stimuli applied simultaneously (Pomeranz et al., 1968; Gokin et al., 1977). Higher proportion of inhibitory responses to visceral stimulation in wakefulness is also suggestive of a weaker overall response to visceral events in this state.

### Diverging the Somatovisceral Convergence

As somatic component of the response is attenuated in sleep while at least one group of cells keeps responding to visceral stimulation, it seems that during sleep visceral information can be utilized easily. However, during wakefulness both types of signals are preserved, so the signal transmitted to the brain will be a mixed one. Is there a way to use the uncovered differences between two groups of responding cells to make sense of such signals? We suggest that a simple excitatory summation of the two types of stimuli would allow both somatic and visceral signals to be used in a meaningful way, provided that the thresholds for these types of signals can be changed between the states of vigilance. Adjustment of the sensory thresholds during transition from wakefulness to sleep is a well-known phenomenon in various sensory modalities including down-regulation of somatosensory signaling in sleep (Mukhametov and Rizzolatti, 1970; Gücer, 1979; Livingstone and Hubel, 1981; Issa and Wang, 2008). Indirect evidence of the adjustment has been provided for the upregulation of the visceral signals propagation in SWS as well, since responses to visceral or vagal stimulation increased during sleep in both the insular and somatosensory cortices (Levichkina et al., 2021; Rembado et al., 2021). Presented data are well compatible with the previous results describing sleep-related and wakefulness-related networks existing in the insular cortex.

**FIGURE 4:**
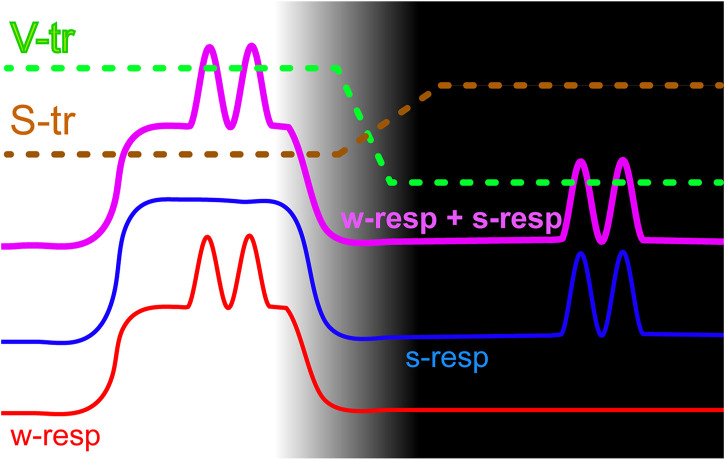
A model of state-dependent signaling that allows separating somatic and visceral types of information by thresholding. Wakefulness and SWS parts of the cycle correspond to the light and dark parts of the picture. The red line schematically shows spiking activity of the wakefulness-responsive (w-resp) cells and the blue line represents sleep-responsive (s-resp) ones. Thick purple line provides spiking rates of a hypothetical cell that receives both types of signals via excitatory inputs. Horizontal sections depict somatic responses while sinusoidally modulated sections show visceral responses. Dashed lines demonstrate thresholds for propagation of somatosensory (S-tr) and visceral (V-tr) signals.

Figure 4 schematically illustrates the proposed model of signal propagation depending on the state of vigilance. It shows patterns of activity of wakefulness-responsive (red line labeled “w-resp”) and sws-responsive (blue “s-resp” line) cell groups in both wakefulness (light part of the figure) and SWS (dark part of the figure). Flat levels of spiking represent a presence/absence of somatic activity, while sinusoidally modulated parts denote visceral responses. As described above, wakefulness-responsive cells have lower spiking rates in both states of vigilance, and in wakefulness they respond to both visceral and somatic simulation. Sleep-responsive ones have only somatic responses during wakefulness, but respond to visceral stimulation selectively in sleep. Solid purple line demonstrates the result of excitatory convergence of both types of stimuli into a hypothetical summation unit. Dashed lines show perceptual thresholds for somatosensory (S-tr) and visceral (V-tr) signals correspondingly.

We presume that perceptual thresholds have opposite dynamics at the transition between wakefulness and sleep. During wakefulness somatosensory threshold is low and somatosensory signals propagate easily, while only very strong visceral signals would add a sufficient amplitude modulation to propagate through the elevated visceral threshold. During sleep somatosensory threshold rises to avoid disturbing the animal’s sleep with unimportant low-level somatosensory signals, while visceral threshold is lowered since “purified” convergent signal now provides useful visceral information, which can serve for regulatory purposes.

One limitation of our study is that it cannot clarify whether it is possible that strong somatosensory stimulation might also propagate in sleep, as that would require the use of somatosensory stimulation which can potentially disturb sleep and wake an animal up. However, if so, the elevated somatosensory threshold (S-tr) during sleep would allow only the strongest signals to propagate, the ones important enough to result in awakening. In an event when strong somatosensory stimulation occurs in sleep the heightened somatosensory threshold would serve as an alarm.

The above described arrangement would result in certain characteristics of perception and sensation, which are well-compatible to the real life observations. Firstly, variability of somatosensory input would prevent the system from deriving detailed and clear visceral signal (suitable for regulation of visceral parameters) during wakefulness. Indeed, the most known effect provided by the convergent signals in wakefulness is a referred pain—relatively crude sensation without spatial clarity, prone to misinterpretation and modulated by various non-visceral stimulation (e.g., rubbing of a painful area or applying pressure to it, postural changes etc.). Somatic component of the input during wakefulness can potentially be improved to some extent by comparing it to the corresponding signals coming through more specific purely somatosensory spinal fibers that exist within the pathway, or even by comparing activities of wakefulness-responsive cells to the ones of the sleep-responsive cells as sleep-responsive ones are purely somatic during wakefulness. Somatic signals of both groups of cells would also be synchronized in time, that can help in separating signal types as synchronized signals can propagate easier (Singer, 1994; Bastos et al., 2015).

The likely aim of the propagating visceral discomfort in wakefulness is to induce behaviors that might be helpful in alleviating it, such as reducing movements, finding an appropriate posture and potentially going to sleep. It is known that sleep facilitates some reparatory processes, especially the ones associated with immune responses (for the reviews see e.g., Benca and Quintans, 1997; Besedovsky et al., 2012; Haspel et al., 2020), while poor sleep quality caused by environmental factors is associated with prolonged recovery and increased mortality in sick people, e.g., in ICU patients (Delaney et al., 2015). Interestingly, based on our results we can also assume better visceral signal clarity in sleep that can potentially help in identifying a source of a problem. The other type of visceral stimuli that is also known to be transmitted via the spinal cord and propagate during wakefulness is the response to excessive distension of distensible organs, which also require only relatively straightforward behavioral responses such as stopping eating, voiding bladder, defecating etc. More specific signals regarding chemical or mechanical visceral signals are transmitted via the vagus nerve that can also have heightened activity during sleep (Leichnetz, 1972; Ramet et al., 1992), accompanied by increased parasympathetic activity (Trinder et al., 2001; Cabiddu et al., 2012).

On the other hand, in order to meet the challenges of active wakefulness it is essential to maintain robust regulation of muscle activity by the brain, which needs to be attenuated in SWS to decrease chances of acting without proper behavioral control of the actions, and to let reparatory process take its course in the muscle tissue. Indeed, sleep is accompanied not just by the changes of the muscle tone, but also by diminished of connectivity within the brain-muscle network. This connectivity is strongest in wakefulness and progressively decreases in REM, light sleep and deep sleep (Bashan et al., 2012; Balagué et al., 2020; Rizzo et al., 2020). Our earlier observations are consistent with this view as electrical stimulation of the insular cortex produced robust muscle response in wakefulness that disappeared as soon as sleep started developing (Levichkina et al., 2021).

We assume that summation of the responses from two groups of cells can contrast the levels of spiking that would make thresholding easier. Annoyingly, nature doesn’t always follow human’s logic, and one cannot guarantee that the summation—thresholding is the way used to distinguish somatic and visceral signals. However, the biggest advantage for the system that needs to extract information is having two different types of responses, as they can be used in several ways to extract information. For example, individual thresholding for each type of cells can take place instead of the summation. Mutual inhibition between the groups of cells to achieve “tip of an iceberg” effect, also scaled by thresholding, is conceivable as well. Discovering which system is actually implemented by the brain is a matter of future studies.

We propose that somatic and visceral neurons across nervous system from the spinal cord to the cortical level can make use of both somatic and visceral signals as long as they possess appropriately “thresholded” neurons that can derive relevant information in the above-described fashion. In fact, one such dual channel simultaneously supplying visceral information to the solitary tract nucleus and somatosensory information to the dorsal column nuclei *via* branching axons, spinosolitary tract-dorsal column postsynaptic neuronal system (SST-DCPS), was found by Lü et al. (2002). Mechanisms of selective regulation of local sleep in different sensory modalities by thalamic reticular nuclei have been described as well (Vantomme et al., 2019).

It is generally accepted that primary and secondary somatosensory cortices existed in early mammals. Although different ecological adaptations led to differences in cortical areas between species, these functional units are present in all mammals (Kaas, 2012). The insular cortex which is often considered as the primary viscerosensory/visceromotor area is also present and has similar organization in many studied placental mammals including rabbits (Nieuwenhuys, 2012). Well-known direct cortical targets of the visceral signals comprise a rather large proportion of cortex that include insula, somatosensory cortices, cingulate, and ventromedial prefrontal areas, while other areas can be influenced indirectly via the hypothalamus (Azzalini et al., 2019). Primate insula underwent great evolutionary expansion, and the primate somatosensory system has specialized as well to accommodate changes in organization of locomotion and, above all, primate reaching and grasping behavior and their developed manual dexterity. Insular cortex also became involved in supporting somatosensory functions, especially the ones related to food manipulation and consumption. Considering expansion of the cortex in primates and especially in humans, it seems especially relevant that insula possess connections with cortical representations of all sensory modalities, and with associative and motor frontal and parietal areas (Öngür and Price, 2000; Saper, 2002; Stephani et al., 2011; Nieuwenhuys, 2012), and comprises an important node of the “medial prefrontal network” of visceral regulation (An et al., 1998; Ongür et al., 1998). That further highlights the possibility of extensive involvement of a large proportion of cortex in the analysis of both somatosensory and visceral signals in humans, with a potential for mutual influences occurring between cortical and visceral dysfunctions.

The presence of the main parts of the sleep-wake cycles such as the wakefulness, SWS and REM in mammals extends to the rabbits as well, and rabbits demonstrate typical for the other studied mammalian species effects of sleep deprivation (Tobler et al., 1990). As summarized in a review by Cirelli and Tononi (2008), “there is no convincing case of a species that does not sleep, no clear instance of an animal that forgoes sleep without some compensatory mechanism, and no indication that one can truly go without sleep without paying a high price.” Prolonged sleep deprivation was shown to provoke multiple visceral disturbances and eventually cause death in rat experiments (Rechtshaffen and Bergmann, 2002), that is unfortunately also true for humans as fatal familiar insomnia syndrome results in severe autonomic dysfunction and leads to death (Lugaresi and Provini, 2007). Our results suggest that somatovisceral convergence, on one hand, can play a role in supplying both somatosensory and visceral types of information but, on the other hand, the system based on such convergence would be prone to errors in situations requiring unusually fast transitions between sleep and wakefulness, such as sleep deprivation, when micro-sleep events are forced to occur in the brain during wakefulness-related behaviors. Signal omissions and misdirections that could result from such errors are likely to add to the pathologies associated with sleep deprivation.

## Conclusion

We found that ascending pathway of the spinal cord, known to express a considerable degree of somatovisceral convergence, contains two groups of cells. One group responds to visceral stimuli selectively during sleep and has predominantly excitatory responses, while the other responds only during wakefulness and shows a considerably larger proportion of inhibitory responses. The differences between these groups also extend to their fiber diameters, spike rates, and spike rate changes across sleep-wake cycle; however both groups demonstrate somatic activity expressed in significant correlation of spike rates with EMG. In addition, pattern of spiking activity in sleep-wake cycle suggests that convergent somatovisceral units keep transmitting information during SWS, despite a decrease of the muscle tone. That is consistent with the hypothesis that somatovisceral cells of the spinal cord collectively provide to the brain mainly somatosensory information in wakefulness and mainly visceral information in sleep. The observed differences in state-related spiking and in response to stimulation allowed us to suggest a way of decoding signals transmitted by the convergent units into somatosensory and visceral types of information by signal thresholding adapted to a state of vigilance.

Our results offer a solution for a very old problem—the functional significance of somatovisceral convergence. The “nonsensical” convergence of two seemingly unrelated signals can actually be an elegant economical way of providing four different signals via the same route, namely, the somatosensory and nociceptive signals in wakefulness, and the regulatory-visceral and awakening-somatosensory ones during sleep.

## Data Availability

The raw data supporting the conclusions of this article can be made available by the authors upon a reasonable request.
